# Cranioplasty With a Customized Polymethylmethacrylate Implant Using a 3D Printer

**DOI:** 10.7759/cureus.50830

**Published:** 2023-12-20

**Authors:** Jahnavi P Gorripati, Surekha A Godbole Dubey

**Affiliations:** 1 Prosthodontics, Sharad Pawar Dental College and Hospital, Datta Meghe Institute of Higher Education and Research, Wardha, IND

**Keywords:** additive manufacturing, rehabilitation, cranial reconstruction, 3d printing, cranioplasty

## Abstract

The occurrence of cranial injuries has increased dramatically, and due to greater awareness among the people concerned with aesthetics needing skull reconstruction, rehabilitation of these defects has also increased in our modern age. Rehabilitation of these deficiencies with prostheses not only works as a protective shell but also improves the patient's neurological state. These cranial deformities necessitate surgical correction or repair, known as cranioplasty. Its goal is not only to rehabilitate the imperfection aesthetically, but also to alleviate psychological issues and improve the patient's social acceptance and performance. New biomedical tools, technologies, and materials are available to surgeons and prosthodontists to improve aesthetics and functions. The purpose is to develop a novel method involving affordable 3D printing technology for creating individualized polymethylmethacrylate (PMMA) implants, aiming to democratize technology in prosthodontics.

## Introduction

Cranioplasty is a surgical procedure that improves the underlying physiology of the brain, including cerebral hemodynamics and metabolism, as well as anatomical restoration [1]. But in those with significant skull deformities, replacement of the skull bone during surgery is not possible, resulting in a significant skull deformity that may necessitate additional cranioplasty, e.g., after decompressive hemicraniectomy. Cranioplasty is routinely performed for mechanical, cosmetic, and physiological reasons. When cranial abnormalities are repaired with this prosthesis, the underlying brain tissue is protected, and the contour of the lost cranial structure is recreated. It also reduces anxiety, eases discomfort at the location, and improves neurological and psychosocial rehabilitation. Osteoplastic reconstruction or replacing it with alloplastic materials such as metals and non-metal silicone are the two methods used to fix these abnormalities. Throughout history, various innovative materials and procedures have been consistently developed or enhanced to address this complex challenge properly [2]. Implants should be inexpensive, easily customized, and readily available during surgery. While autologous bone is still the preferred option for restoration, its usage is not always possible because of infection, fragmentation, resorption of the bone, or other reasons [3]. As a result, synthetic substitutes such as metals, ceramics, plastics, resorbable polymers, and biomaterials are used. Polymethylmethacrylate (PMMA) is the most commonly utilised allogenic material with long-term success for cranial reconstructions [4]. Originating as an industrial material, Zander repurposed it for human calvarial repair in 1940, and Gurdjian et al.'s published work popularized it shortly after. Its advantages included biocompatibility, affordability, strength, and moldability. The advancement of cranioplasty has coincided with technological advancement, the expansion of our collective imagination, and our goal of achieving maximum benefit with the least risk and environmental impact. The purpose is to develop a novel method involving affordable 3D printing technology for creating individualized PMMA implants, aiming to democratize technology in prosthodontics

## Case presentation

**Figure 1:**
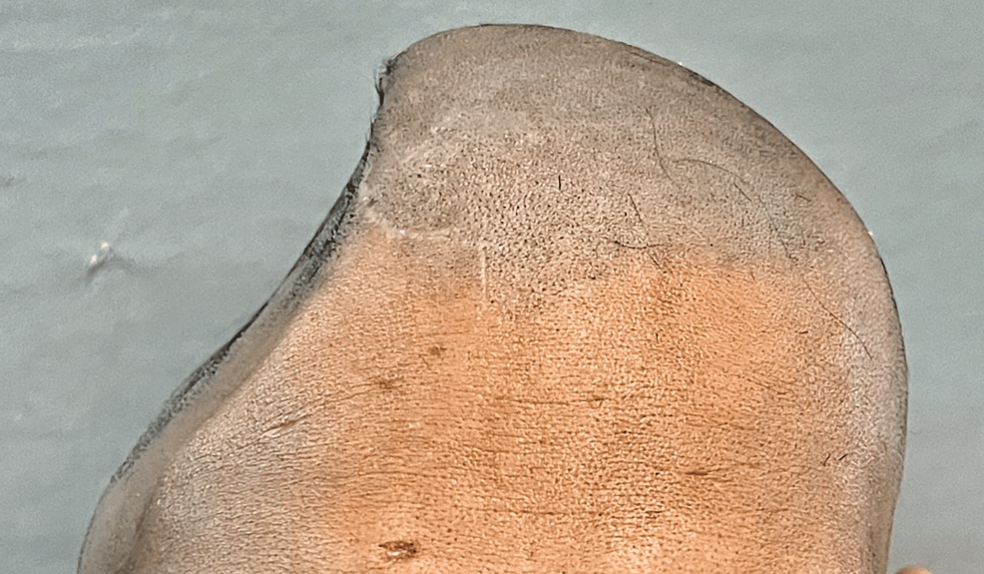
Patient photographs frontal view of defect area before reconstruction

**Figure 2:**
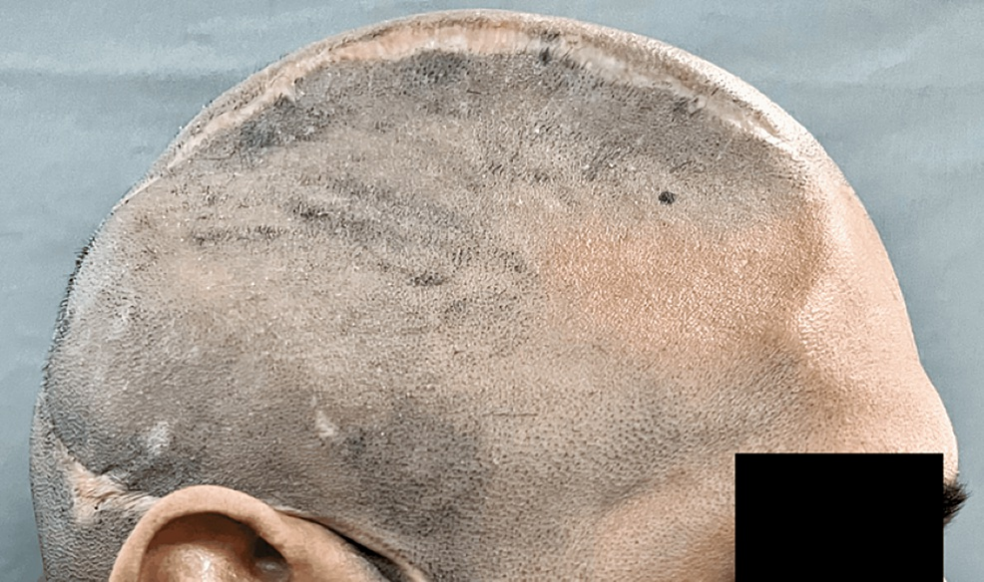
Patient photographs lateral view of defect area before reconstruction

A 38-year-old man who was previously traumatized in a car accident that resulted in several bone fractures, including the cranium. The patient's primary complaint, a postoperative calvarial deformity, led to the patient being directed to the Department of Prosthodontics to manufacture the cranial prosthesis. The right frontal, parietal, and temporal bones were affected by the deformity. The flaw was 16 cm by 9 cm by 6 cm in size (Figures 1, 2).

**Figure 3:**
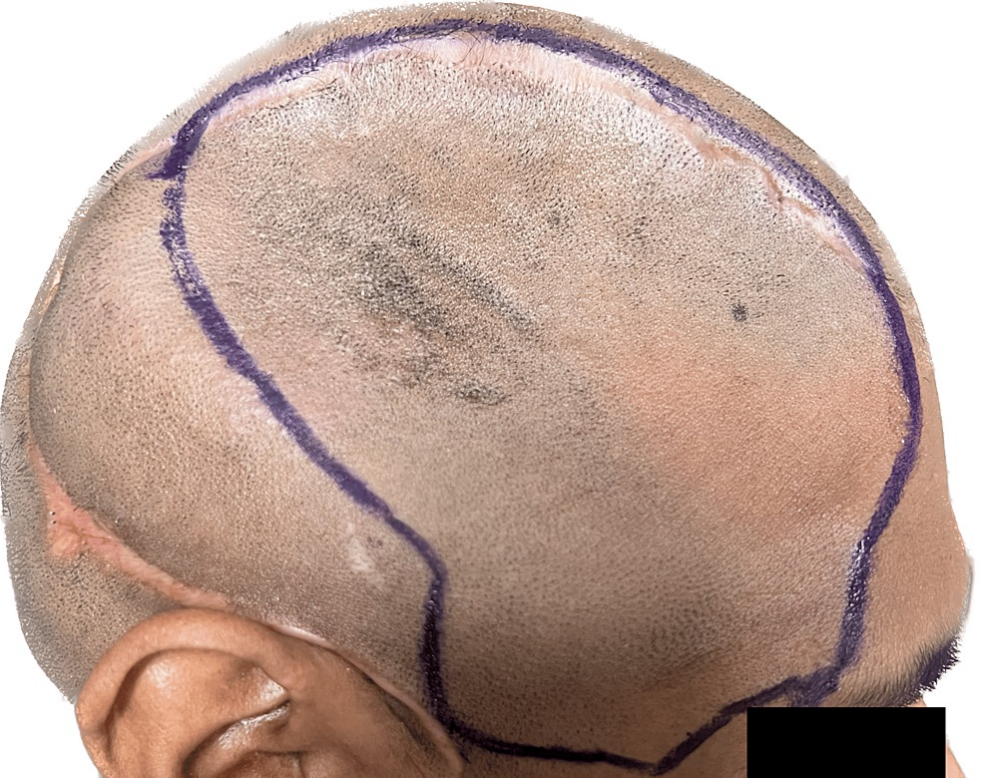
Outline the defect using indelible pencil by palpating the bony edges

**Figure 4:**
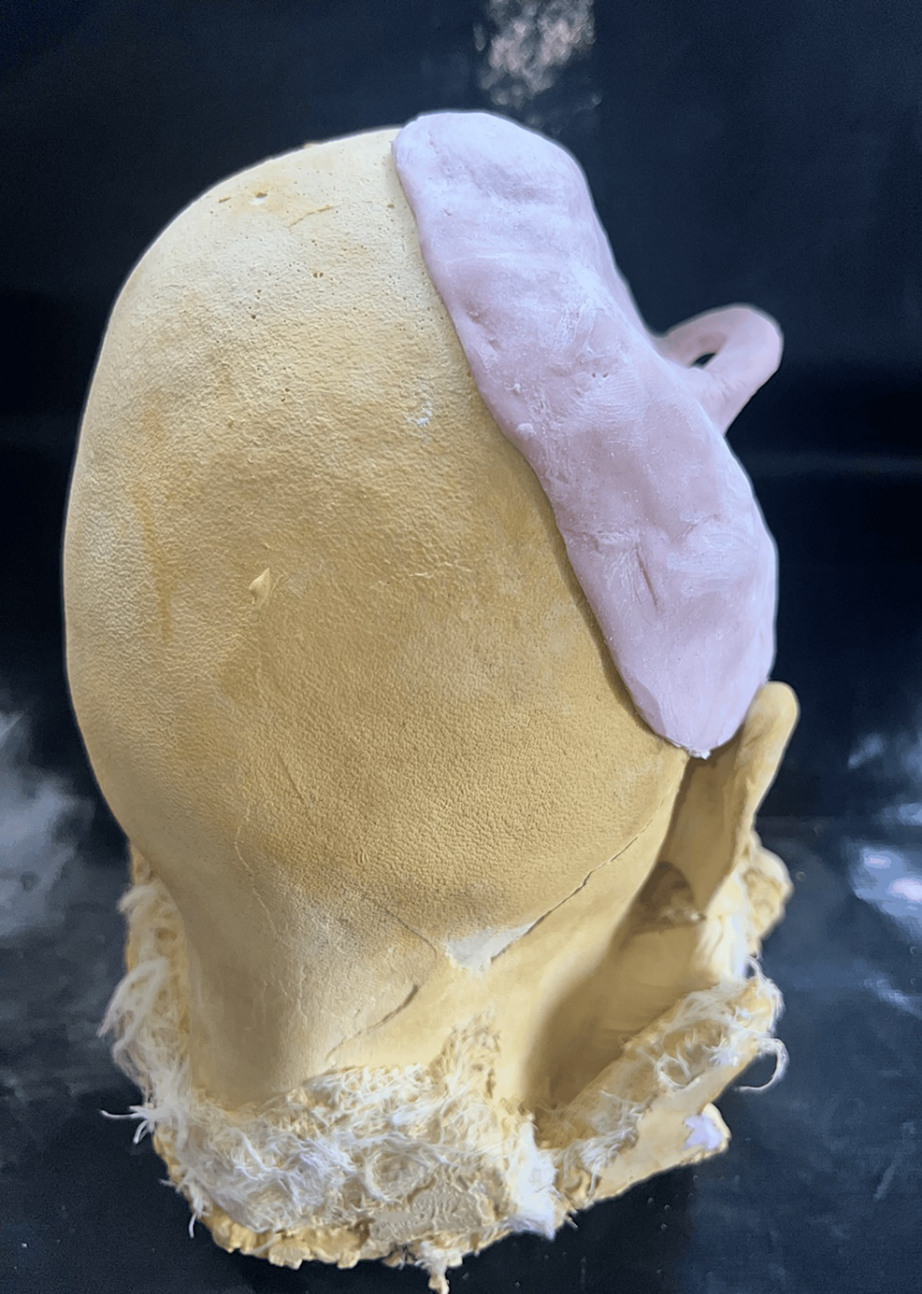
Custom tray with handle made up of tray material on primary cast

Prosthetic and surgical phases encompassed the process of making cranial prosthesis fabrication. During the prosthetic phase, a face moulage was made using an irreversible hydrocolloid impression material and prior to that, an indelible pencil is used to outline the defect by palpating the bony boundaries (Figure 3). Since it was challenging to capture the irregular defect margins in the frontal and temporal bones in the face moulage, a custom tray was made utilizing the original cast that was obtained (Figure 4).

**Figure 5:**
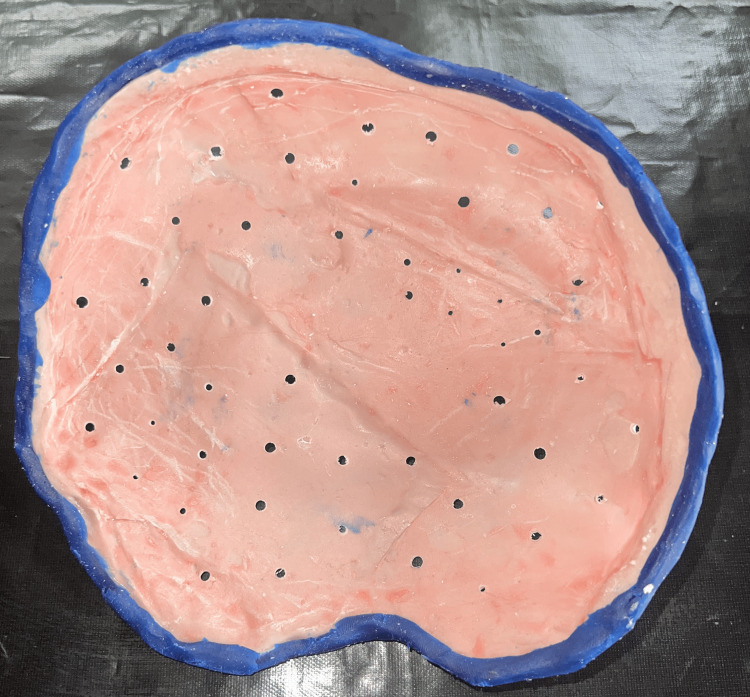
Custom tray beaded with beading wax to confirm the impression materials

**Figure 6:**
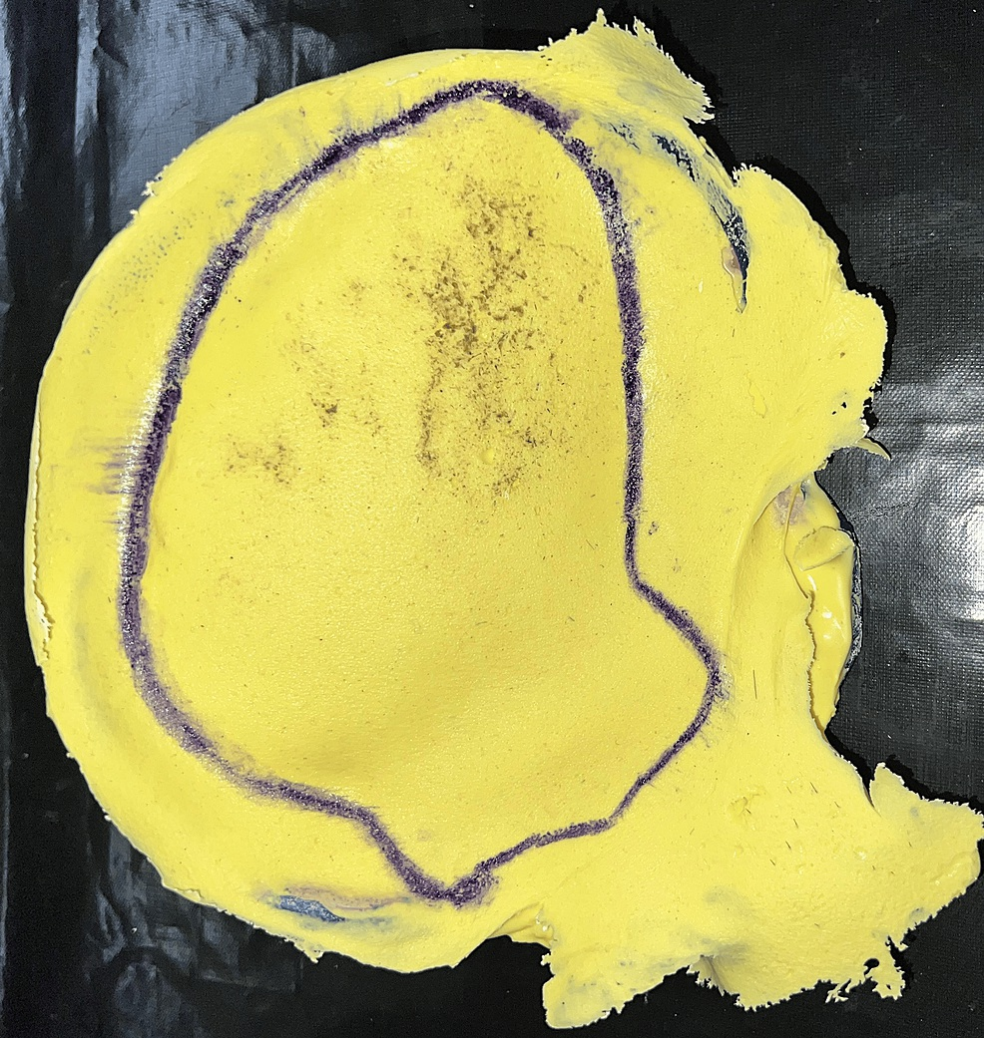
Final impression of the defect

Beading wax was used to line the custom tray containing and controlling the material while highlighting any defects and impressions made (Figures 5, 6).

**Figure 7:**
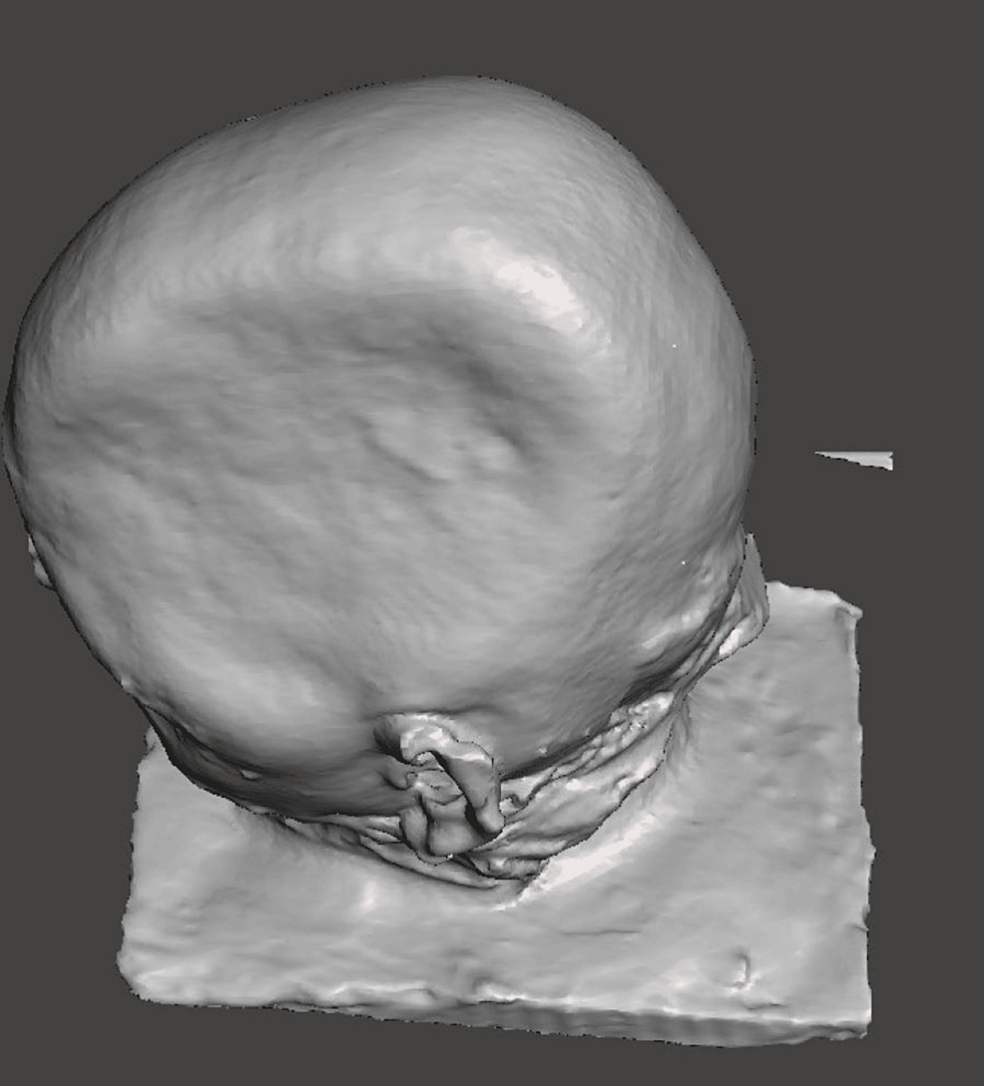
CT scan of the primary cast with cranial defect margins

**Figure 8:**
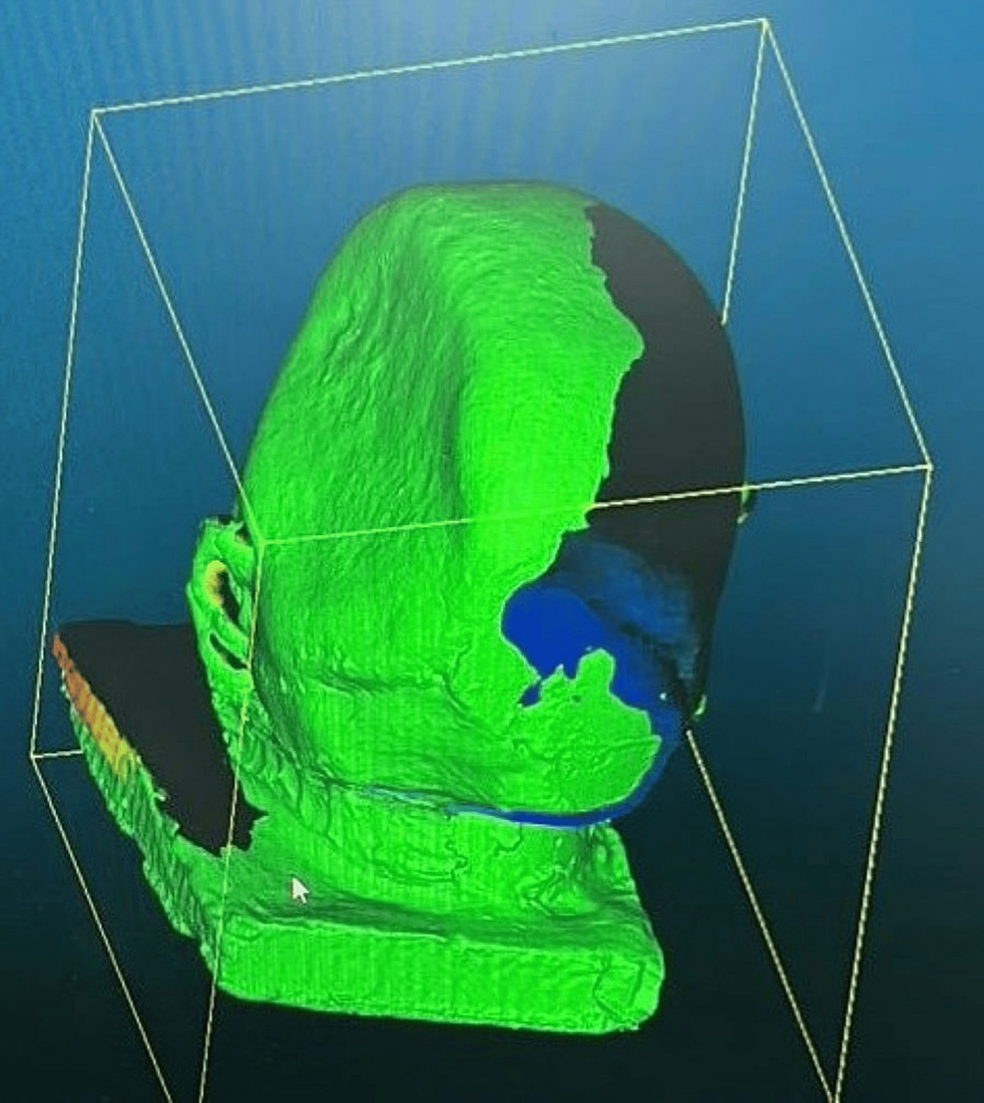
Scanned file of the cranial defect using extra oral scanner

Gypsum products classified as Type III are used to create the working cast. A CT scan of the primary cast was made to get the accurate margin of the cranial defect (Figure 7). To create a design and 3D-printed cranial prosthesis, the cast was scanned using an extra-oral scanner (Figure 8).

The bilateral symmetry of the 3D-printed cranial prosthesis was verified using a trail. Simultaneously, an overall thickness of 3 mm is preserved, and the PMMA cranial prosthesis's edges are beveled to 45 degrees by which a snug fit with a bone is obtained. Digital 3D printing was used to create the PMMA cranial prosthesis [4], which was then checked on a stone replica. Following verification, it was used intraoperatively, cleaned, and kept for around 24 hours in a 2% glutaraldehyde solution. The prosthesis's perforations facilitated the removal of inflammatory exudates and supplied blood to the flap tissues that overlay them, primarily the scalp. The prosthesis's smooth dorsal surface makes it presentable and enables for a correct fit.

**Figure 9:**
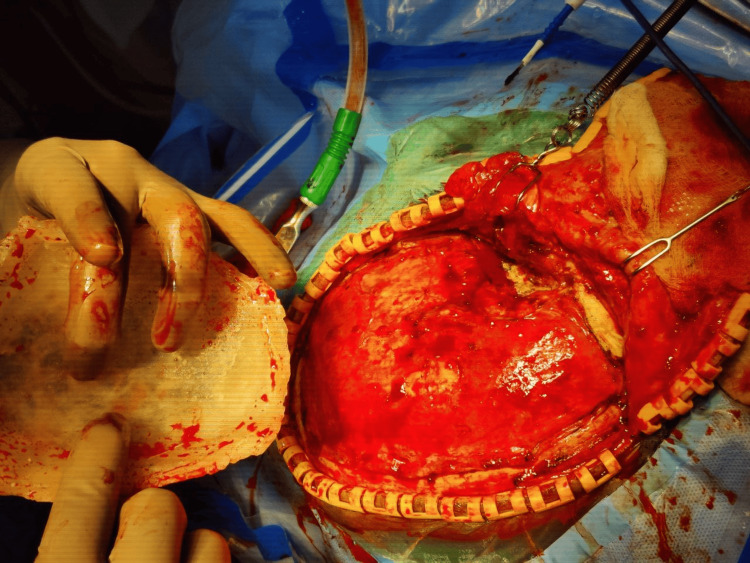
Surgical phase: exposure of the defect placement

**Figure 10:**
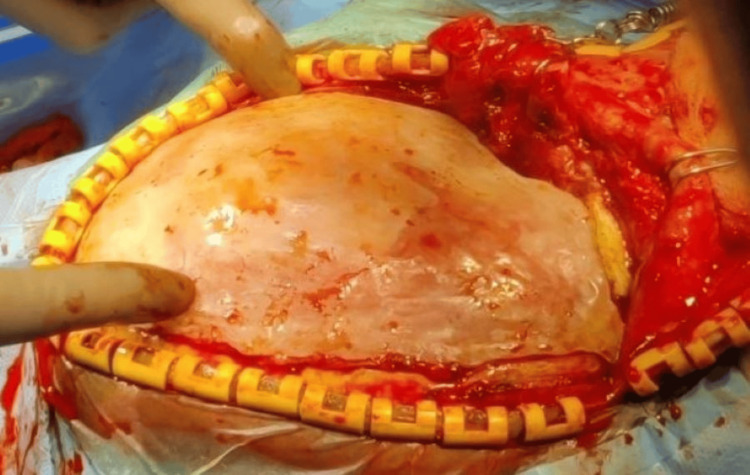
Fixation of the 3D printed polymethyl methacrylate cranial prosthesis

The implantation procedure was conducted in compliance with the guidelines set by the institution. Along the prior incision, skin was opened, and then the bony borders, (neo-)dura, and temporalis muscle flap were prepared (Figure 9). Four four-hole plates and self-tapping titanium screws were used to secure the implants (Figure 10).

**Figure 11:**
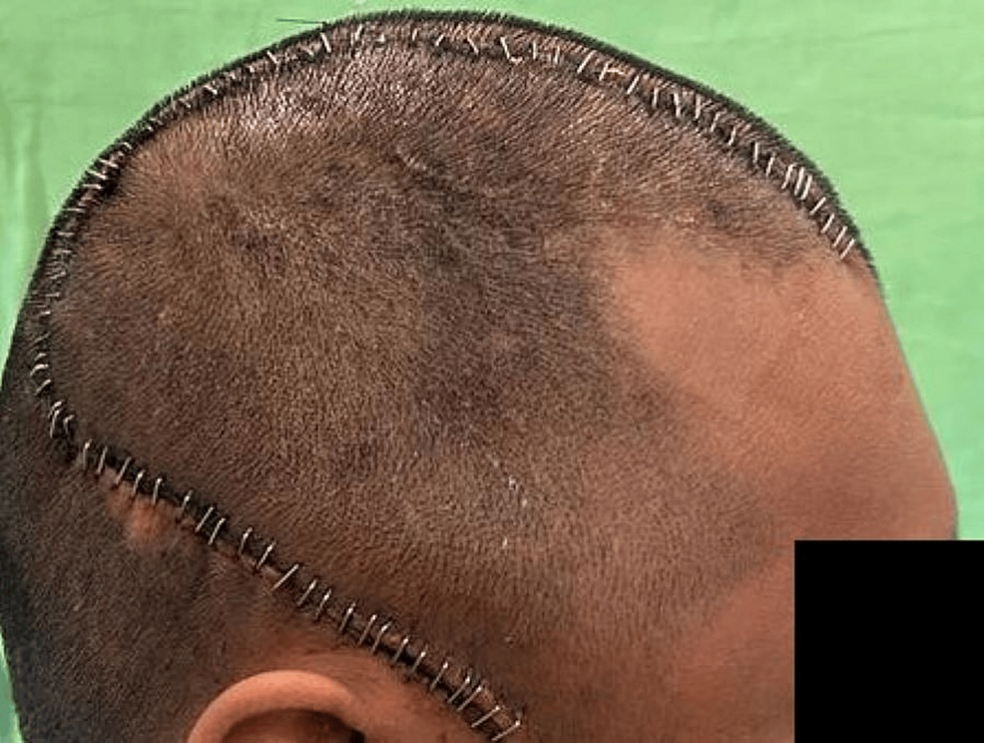
Post surgical picture after cranioplasty reconstruction

Many resorbable sutures for retention were used to connect the dura to the implant in order to prevent the accumulation of epidural fluid. After the insertion of one or two subgalea drains with suction, the wound was closed. After one week of surgery, the patient was evaluated in the prosthodontics department, and the patient's skull contour had significantly improved (Figure 11). The patient expressed great satisfaction with aesthetics he received, as well as the relief from his terrible headache and psychological anguish. After four months, the patient was evaluated and was going about his social life as usual.

## Discussion

Cranioplasties have been carried out since the early 1950s [5]. The field of cranioplasty has advanced steadily. Much progress has been made in the last 20 years in studying the methods used to create personalized cranioplasty implants [6,7]. However, these remarkable methods have stayed in the different research facilities and academic institutions where they were created.

This is because they require specialized knowledge and expensive, sophisticated equipment and software that is out of reach for most hospitals and surgeons. As a result, not all patients needing cranioplasty have profited from advancements in the field. Outside of academia, for-profit businesses provide these services; however, they typically charge a premium fee for them. Cranial decompression is the most utilized treatment for elevated intracranial pressure caused by severe injuries, cerebral infarctions or haemorrhage, tumors, and other causes. Aside from aesthetics [6], repair of these anomalies stabilises the patient's hemodynamic functioning, neurological status, and psychosocial status.

Dean et al. [8] found that PMMA or pre-bent titanium cranioplasty implants made with CT data were more aesthetically pleasing and better fitting than prepared implants; additionally, they found that these snug-fit implants were more likely to shield the brain from injury and infection. Goh et al. [9] found that utilising customised implants made with computer-aided design helps to prevent unsatisfactory surgical outcomes and ought to be the recommended course of action.

The most common method for restoring lost cranial bone is alloplastic cranioplasty using PMMA. These processes require that the plastic be modelled while it is polymerising. The cosmetic effect can be difficult because of the curved shape of the skull [7]. The difficulty of freely designing a large PMMA plastic in surgical situations is one of the main problems. Thus, designing and producing customised implants has advanced from free-hand moulding to computer-assisted moulding in just a few decades. Although the accessibility of 3D printing has increased, little attention has been paid to how open-source, affordable software and a desktop three-dimensional printer have the potential to enhance prosthodontics globally and democratise technology [10]. Anyhow, we want to contribute to the advancement of customised cranioplasty by offering our knowledge of a reliable and affordable method that any organisation using a desktop 3D printer and open-source or inexpensive software can use [9]. Ultimately, every creative and innovative mind will benefit from the democratisation of technology as it helps to extend and move the world in new ways. The limitations of the present technique is use of the software for multiple cases as the subscription has to be taken which is expensive. The future scope is there is a requirement for study to be done in large populations to get accurate results. 

## Conclusions

A novel method has been designed for generating and fabricating sterile, three-dimensional printed templates, which are subsequently utilised to manufacture individualised PMMA implants for cranioplasty procedures. The most perfect rebuilding of pre-craniectomy skull form has been accomplished by focusing on the external surface of the implant; this method has been shown to be cost-effective due to use of open-source software (for 48hrs) and user-friendly. When the method is applied clinically, great cosmetic outcomes are produced, and the designing and printing are repeated.
